# Development of (NO)Fe(N_2_S_2_) as a Metallodithiolate Spin Probe Ligand: A Case Study Approach

**DOI:** 10.1021/acs.accounts.3c00667

**Published:** 2024-02-28

**Authors:** Manuel Quiroz, Marcetta Y. Darensbourg

**Affiliations:** Department of Chemistry, Texas A & M University, College Station, Texas 77843, United States

## Abstract

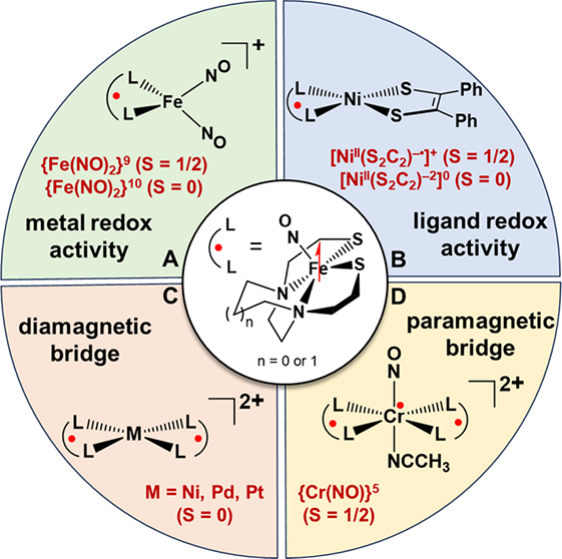

The ubiquity of sulfur–metal connections in nature inspires the design of bi- and multimetallic systems in synthetic inorganic chemistry. Common motifs for biocatalysts developed in evolutionary biology include the placement of metals in close proximity with flexible sulfur bridges as well as the presence of π-acidic/delocalizing ligands. This Account will delve into the development of a (NO)Fe(N_2_S_2_) metallodithiolate ligand that harnesses these principles. The Fe(NO) unit is the centroid of a N_2_S_2_ donor field, which as a whole is capable of serving as a redox-active, bidentate S-donor ligand. Its paramagnetism as well as the ν(NO) vibrational monitor can be exploited in the development of new classes of heterobimetallic complexes. We offer four examples in which the unpaired electron on the {Fe(NO)}^7^ unit is spin-paired with adjacent paramagnets in proximal and distal positions.

First, the exceptional stability of the (NO)Fe(N_2_S_2_)-Fe(NO)_2_ platform, which permits its isolation and structural characterization at three distinct redox levels, is linked to the charge delocalization occurring on both the Fe(NO) and the Fe(NO)_2_ supports. This accommodates the formation of a rare nonheme {Fe(NO)}^8^ triplet state, with a linear configuration. A subsequent FeNi complex, featuring redox-active ligands on both metals (NO on iron and dithiolene on nickel), displayed unexpected physical properties. Our research showed good reversibility in two redox processes, allowing isolation in reduced and oxidized forms. Various spectroscopic and crystallographic analyses confirmed these states, and Mössbauer data supported the redox change at the iron site upon reduction. Oxidation of the complex produced a dimeric dication, revealing an intriguing magnetic behavior. The monomer appears as a spin-coupled diradical between {Fe(NO)}^7^ and the nickel dithiolene monoradical, while dimerization couples the latter radical units via a Ni_2_S_2_ rhomb. Magnetic data (SQUID) on the dimer dication found a singlet ground state with a thermally accessible triplet state that is responsible for magnetism. A theoretical model built on an H_4_ chain explains this unexpected ferromagnetic low-energy triplet state arising from the antiferromagnetic coupling of a four-radical molecular conglomerate. For comparison, two (NO)Fe(N_2_S_2_) were connected through diamagnetic group 10 cations producing diradical trimetallic complexes. Antiferromagnetic coupling is observed between {Fe(NO)}^7^ units, with exchange coupling constants (*J*) of −3, −23, and −124 cm^–1^ for Ni^II^, Pd^II^, and Pt^II^, respectively. This trend is explained by the enhanced covalency and polarizability of sulfur-dense metallodithiolate ligands. A central paramagnetic *trans*-Cr(NO)(MeCN) receiver unit core results in a cissoid structural topology, influenced by the stereoactivity of the lone pair(s) on the sulfur donors. This {Cr(NO)}^5^ radical bridge, unlike all previous cases, finds the coupling between the distal Fe(NO) radicals to be ferromagnetic (*J* = 24 cm^–1^).

The stability and predictability of this *S* = 1/2 moiety and the steric/electronic properties of the bridging thiolate sulfurs suggest it to be a likely candidate for the development of novel molecular (magnetic) compounds and possibly materials. The role of synthetic inorganic chemistry in designing synthons that permit connections of the (NO)Fe(N_2_S_2_) metalloligand is highlighted as well as the properties of the heterobi- and polymetallic complexes derived therefrom.

## Key References

- Ghosh, P.; Ding, S.; Quiroz, M.; Bhuvanesh, N.; Hseih, C.-H.; Palacios, P. M.; Pierce, B. S.; Darensbourg, M. Y.; Hall, M. B. Structural and Electronic Responses to the Three Redox Levels of Fe(NO)N_2_S_2_–Fe(NO)_2_. *Chem. Eur. J*. **2018**, *24*, 16003–16008.^1^ The stable diradical [(NO)Fe(N_2_S_2_)-Fe(NO)_2_]^+^ shows strong antiferromagnetic (AFM) coupling between the two irons (at 2.71 Å), resulting in diamagnetism. The (NO)Fe(μ-SR)_2_Fe(NO)_2_ connectivity is maintained over three redox levels, with vibrational spectroscopy, magnetism and solid state structures tracking the fate of added electrons.
- Quiroz, M.; Lockart, M.; Saber, M.; Vali, S. W.; Elrod, L. C.; Pierce, B. S.; Hall, M. B.; Darensbourg, M. Y. Cooperative Redox and Spin Activity from Three Redox Congeners of Sulfur-bridged Iron Nitrosyl and Nickel Dithiolene Complexes. *Proc. Natl. Acad. Sci. U.S.A*. **2022**, *119* (25), e2201240119.^2^ Monomeric [Fe–Ni]^+^ exhibits AFM coupling (*J* ≈ −1200 cm^–1^) between (NO)Fe(N_2_S_2_) and the radical dithiolene on nickel. In the dimeric form [Fe(Ni_2_S_2_)Fe]^2+^, a diamagnetic Ni_2_S_2_ bridge facilitates magnetic coupling (*J* = −54 cm^–1^) between two (NO)Fe(N_2_S_2_) 8 Å apart.
- Quiroz, M.; Lockart, M.; Xue, S.; Jones, D.; Guo, Y.; Pierce, B. S.; Dunbar, K. R.; Hall, M. B.; Darensbourg, M. Y. Magnetic Coupling between Fe(NO) Spin Probe Ligands through Diamagnetic Ni^II^, Pd^II^ and Pt^II^ Tetrathiolate Bridges. *Chem. Sci.***2023**, *14*, 9167–9174.^3^ Transoid structures of [(NO)Fe(N_2_S_2_)]_2_-M^2+^ have two Fe(NO) radical units at 6 Å. Superexchange via the bridging thiolates and central metal exhibit *J* values of −3, −23, and −124 cm^–1^, reflecting enhanced covalency in M–S bonds [3*d* (Ni) < 4*d* (Pd) < 5*d* (Pt)].
- Guerrero-Almaraz, P.; Quiroz, M.; Rodriguez, D. R.; Jana, M.; Hall, M. B.; Darensbourg, M. Y. The Uncommon Isomer: Sulfur-lone pairs control topology and a hydrocarbon-lined pocket in heterotrimetallic trans-Cr(NO)L[M(N_2_S_2_)]_2_ complexes. *ACS Org. Inorg. Au***2023**, *3*, 393.^4^ A central *trans*-Cr(NO)(MeCN) radical unit positions two (NO)Fe(N_2_S_2_), *S* = 1/2 metalloligands in a cissoid structure 6 Å apart. The π-withdrawing ability of NO stabilizes the unfavorable electrostatic repulsions of four S lone pairs lying underneath a hydrocarbon-lined pocket.

## 1 Introduction

Diatomic molecules that interact with iron are fundamental to life on planet earth. These include O_2_, CO, H_2_, N_2_ as well as NO. Nitric oxide is pivotal in the nitrogen cycle, acting as an essential intermediate compound that aids in the conversion of atmospheric nitrogen into forms such as nitrate and nitrite, enabling its utilization by plants and ecosystems.^5−8^ Nitric oxide is also physiologically important to humans as a signaling agent, and it plays a pivotal role in vascular regulation and immune response, largely involving iron-hemes as a vehicle for its transport and activity.^9−12^ The Fe(NO) unit appears in the isolated active site of nitrile hydratase (NHase), an enzyme that facilitates hydration of nitriles.^13^ Therein a Cys-Ser-Cys dianionic tripeptide motif provides a N_2_S_2_ binding site that has been post-translationally modified to be “softened” by sulfur oxygenation, rendering the S-donors less nucleophilic but positioning sulfenate or sulfinate oxygens so as to assist in H_2_O addition to metal-bound nitriles. The cobalt version of NHase does not find NO bound to Co; whether its absence reflects the biosynthetic path or the instability of the Co(NO) unit as compared to Fe(NO) is, to our knowledge, not currently known.^14−16^ Notably the Fe-containing enzyme is activated by light wherein NO is removed and replaced by substrate.^17−19^

Our work with MN_2_S_2_ as bidentate S-donors to exogenous metals led to intricate structural molecular architectures, including with (NO)Fe(N_2_S_2_), finding in it exceptional physical properties to be exploited in the characterization of resultant bi- and polymetallic complexes.^20,21^ The (NO)Fe(N_2_S_2_) metallodithiolate ligand has been characterized as a bidentate ligand when bound to a redox stable or “innocent” receptor group. The formulations of the resulting complexes are generally consistent with those of diphosphine or bipyridine ligands. Such receivers as [W(CO)_4_]^0^ or [CpFe(CO)]^+^ with two sites for bidentate donor additions have been used to characterize the electronic effects of modifications on the N to N and N to S platform via changes in the ν(CO) reporter.^22−24^ These are typically minor, ranging from open chain N to N connectors to the mesodiazacycles which we have used extensively. The steric rigidity of the latter enforces more stable metallodithiolate derivatives. Other N_2_S_2_ platforms, for example, by Duboc et al., have demonstrated how preorganized ligand structures control complex nuclearity by using bulky phenyl substituents on the carbon atoms adjacent to the sulfur sites. It has been shown that inclusion of a redox noninnocent bipyridine unit as the N to N backbone stabilizes different metal oxidation states.^25,26^

The ν(NO) vibrational mode is valued as a probe of electron density drained from the {Fe(NO)}^7^ unit (in Enemark–Feltham notation where the superscript accounts for the number of metal *d* and NO π* electrons without assignment oxidation states) when it binds to an exogenous metal. Its ability to undergo redox changes from {Fe(NO)}^7^ (*S* = 1/2) to {Fe(NO)}^8^ (*S* = 1) introduces electrochemical and EPR characterization as well. The X-ray diffraction analysis of isolated crystals reveals a typical square pyramidal geometry, where displacement of the Fe(NO) unit out of the N_2_S_2_ plane varies with several factors, including the ∠S–Fe–S, the ∠Fe–N–O, and the τ_5_ value. Figure S1 lists these experimental parameters for a series of nitrosylated iron metallodithiolates.^27−34^

**Figure 1:**
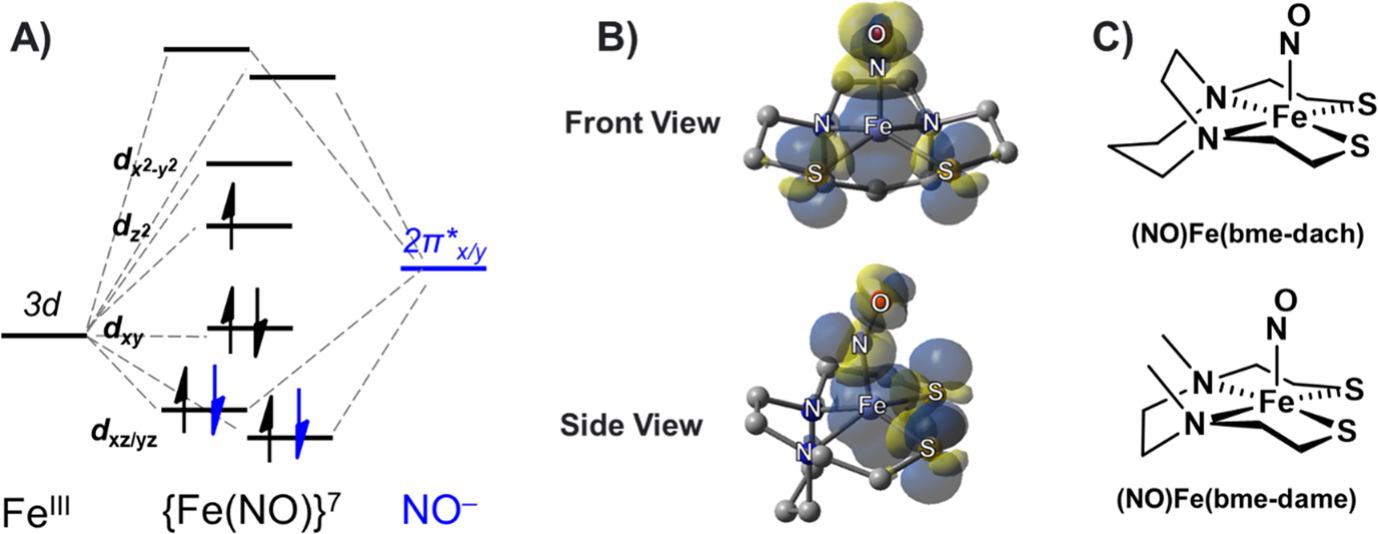
(A) Qualitative molecular orbital diagrams for the (NO)Fe(bme-dach) complex. Black arrows are metal-based electrons, while blue arrows are nitrosyl based. (B) Spin density plots of (NO)Fe(bme-dach) showcasing signification spin delocalization on to the *cis*-dithiolates (isovalue 0.004). Adapted with permission from ref (3). Copyright 2023 Royal Society of Chemistry. (C) The two (NO)Fe(N_2_S_2_) metallodithiolate ligands of the case studies.

Early analyses of MNO assumed a strong correlation between M–N–O angles and the NO oxidation level. Linear was purported to relate to NO^+^ as a 10 electron species analogous to CO, while strongly bent as in Co(NO), typically around 120°, was indicative of NO, analogous to the end on binding of O_2_. In the case of the intermediate values such as typically found in Fe–N–O of ca. 150°, the assumption was that with radical-like character, ^•^NO was operative. To address this assumption, Solomon *et al.* used sulfur K-edge XAS, Mössbauer spectroscopy and extensive DFT studies to establish the electronic characteristics of (NO)Fe(N_2_S_2_) in a structure with ∠Fe–N–O ≈ 153° and ν(NO) ≈ 1655 cm^–1^. The conclusion from these studies is that an *S* = 3/2 Fe^III^, antiferromagnetically coupled to an *S* = 1, NO, best accounts for an overall spin state of *S* = 1/2.^35,36^ A qualitative molecular orbital diagram is shown in Figure 1A, and one can see that the four electrons involved in the π manifold (*d*_*xz*_-π_x_* and *d*_*yz*_-π_y_*) are essentially equally distributed between the iron and nitrosyl, with an unpaired electron having *d*_*z*_^2^ orbital character. As clearly illustrated in Figure 1B by the spin density plots of (NO)Fe(bme-dach) (bme-dach = *N*,*N*-bis(2-mercaptoethyl)-1,5-diazacycloheptane), significant spin density from the unpaired electron on iron is polarized to the *cis*-dithiolates. Hence, heterobimetallic complexes incorporating the (NO)Fe(N_2_S_2_) metallodithiolate as a donor ligand hold promise as compelling probes to investigate the feasibility of electron spin coupling with external metals and to delineate the factors governing such magnetic coupling interactions.

In pursuit of expanding the knowledge database of thiolate bridged bi/polymetallic complexes, we delved further into this redox- and spin-active metallodithiolate ligand. The four Lewis acid electrophiles that have served as building blocks for developing the (NO)Fe(N_2_S_2_) redox and spin active donor in bi- and polymetallic complexes are shown in the Conspectus figure.

## 2 Redox Levels of the (NO)Fe(N_2_S_2_)-Fe(NO)_2_ Platform (Case A)

**Figure 2:**
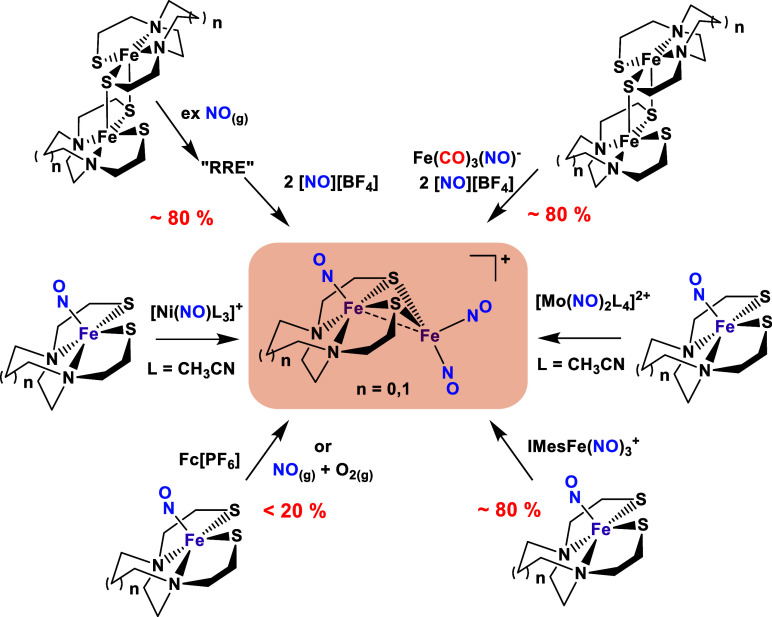
Various pathways to generate the Fe_2_(NO)_3_ cationic complexes from Hsieh et al. Isolated yields of the product were obtained for each pathway; except for the 3 and 9 o’clock paths, the products were observed via IR spectroscopy. Adapted from with permission from ref (1). Copyright 2018 John Wiley and Sons.

The thiolate bridged Fe_2_(NO)_3_ complex shown in Figure 2 can be viewed as a unique model of the 2Fe subsite in the [FeFe]-H_2_ase. It demonstrates cooperative structural changes upon sequential reductions, involving orbital adjustments and fine-tuning of electron density redistribution.^1,37^ Existing in a thermodynamic valley, it forms through various synthetic methods that generate the Fe(NO)_2_ or dinitrosyl iron complex (DNIC) receiver unit. As Figure 2 illustrates, the diamagnetic Fe_2_(NO)_3_ cation, regardless of the N_2_S_2_ [N_2_S_2_ = *N*,*N*′-dimethyl-*N*,*N*′-bis(2-mercaptoethyl)ethylenediamine (bme-dame) or *N*,*N*-bis(2-mercaptoethyl)-1,5-diazacycloheptane (bme-dach)] connectivity in the (NO)Fe(N_2_S_2_) precursor, can be obtained by at least six synthetic approaches even under ambient air conditions.

**Figure 3:**
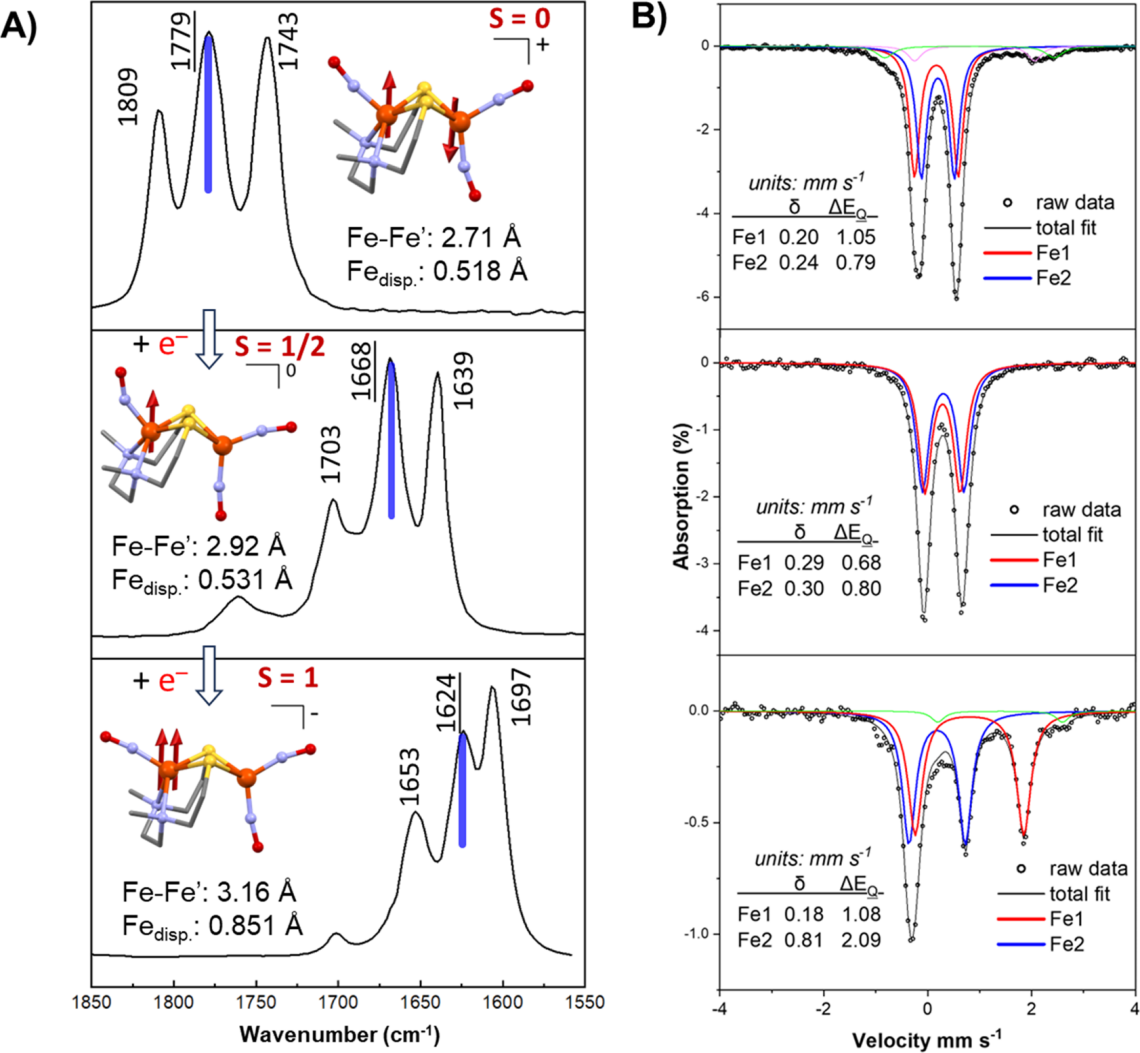
(A) IR spectrum of the sequential reduction of the [Fe*Fe’]^+^ complex to its neutral and anionic forms. Insets show XRD structures with metric parameters and spin states (arrows are shown to illustrate spin centers). (B) Zero-field Mössbauer spectra (dots) and spectral simulations (lines) at 5 K of the [Fe*Fe’]^+/0/–^ series (top, cation; middle, neutral species; bottom, anion).

Convincing arguments are made that DNICs are the likely working form of NO *in vivo* due to enhanced stability within the highly electron-delocalized {Fe(NO)_2_}^9^ and {Fe(NO)_2_}^10^ redox levels.^12,38,39^ DNICs are typically tetrahedral Fe(NO)_2_ units, with donor ligands such as thiolates and imidazoles filling the four coordination sites. As such they are considered for therapeutic use in conditions requiring elevated NO levels, such as diabetic wound healing and asthma treatment, due to their stability and NO-releasing capabilities. Recognizing the ability of iron-nitrosyl units to buffer electronic charge, it was targeted as a receiver unit to exploit its fully reversible {Fe(NO)_2_}^9/10^ redox couple. In fact, this [Fe_2_(NO)_3_]^+^ complex showcased modest electrocatalytic activity for the hydrogen evolution reaction (HER) at the first reduction event. Our further exploration into this paradigm uncovered an intriguing interplay of electrons and structural changes orchestrated by the bridging thiolate sulfurs and nitrosyl ligands. These ligands guided electrons, first toward the more delocalized dinitrosyl iron unit ({Fe(NO)_2_}^9/10^ couple), followed by the second (at more negative potentials) electron’s uptake at the mononitrosyl iron site ({Fe(NO)}^7/8^ couple). These electron positions were revealed by isolation of the species produced and FTIR, EPR and Mössbauer spectroscopies and single crystal XRD analyses (Figure 3).

The crux of our findings lies in the S-bridge itself—a connection that gives, in the cationic form of [Fe_2_(NO)_3_]^**+**^, a 2.71 Å separation between the two iron units, as depicted in the insets of Figure 3A. The dithiolate bridge created a low-spin configuration for the cationic diradical {Fe(NO)}^7^-{Fe(NO)_2_}^9^ due to strong antiferromagnetic coupling between the units and displayed a DFT calculated *J* value of −2650 cm^–1^. Subsequent reductions increase the Fe–Fe separation while preserving the framework. In the two-electron-reduced species with *S* = 1, the Fe(NO) center of the N_2_S_2_ metalloligand showed a notable lift of over 0.3 Å from the N_2_S_2_ plane compared to the one-electron-reduced species and led to a linearized Fe–N–O structure.

From Mössbauer spectroscopy (Figure 3B), a broad doublet is observed for both the cation and the neutral species in which each iron center can be fitted with two individual doublets from the {Fe(NO)}^7^ and {Fe(NO)_2_}^10^ units. The average isomer shift value of 0.22 mm/s in the cationic form increases upon reduction to 0.295 mm/s. The small change suggests that the first electron is added to the {Fe(NO)_2_},^9^ forming {Fe(NO)_2_}^10^, consistent with the report of Neese et al., on a related [Fe(NO)_2_(nacnac)] (nacnac = 1,3-diketimines) system.^40^ The small change is attributed to the strong π back bonding of the iron to the buffering ability of the two nitrosyls. Frozen solutions of the *in situ* generated anionic form exhibit three distinct peaks with varying intensities, which were fitted with two quadrupole doublets corresponding to two distinct iron sites. For one of these doublets, the isomer shift (IS, δ) value remained consistent within the range typically associated with a DNIC (δ = 0.19 mm/s). However, the second doublet exhibited a notable increase in the IS value, reaching 0.81 mm/s. This difference in isomer shift values is reminiscent of the difference seen in Fe^III^ to Fe^II^ complexes.^41^ In this regard, reduction of the {Fe^III^(NO^–^)}^7^ units yields a triplet {Fe(NO)}^8^ from the antiferromagnetic coupling of high-spin Fe^II^ (*S* = 2) and high-spin (*S* = 1) NO^–^. This HS {Fe(NO)}^8^ unit as identified in the [Fe_2_(NO)_3_]^−^ platform is rare and seemingly the first structurally characterized instance of such a unit, evidently stabilized within a binuclear system.

Vibrational spectroscopy analysis shows that the distinctive three-band pattern of ν(NO) undergoes a collective shift of approximately 100 cm^–1^ when the first electron is introduced, followed by an additional shift of around 50 cm^–1^ upon the addition of the second electron. Notably each band is shifted to the same extent! These shifts are primarily attributed to electron delocalization, characterized by a push–pull effect across the sulfur bridges. This result highlights the exceptional capacity of the Fe_2_(NO)_3_ platform to effectively distribute charge over the entire scaffold, despite the loci of the added electrons. The project described in the next section was developed to further separate electrons.

## 3 Cooperative Redox and Spin Activity in Sulfur-Bridged Iron Nitrosyl and Nickel Dithiolene Complexes (Case B)

The design of a bimetallic complex with the same strategy for placing redox activity on the (NO)Fe(N_2_S_2_) donor as well as an adjacent redox active receiver came available with Donahue’s development of a labile-ligand, nickel dithiolene synthon.^42−44^ Thus, the expectation of enlarging the paramagnetic centers from the ca. 3 Å separation in the [Fe_2_(NO)_3_]^+/0/–^ scaffold to systems that have the loci of the adjacent unpaired electron on the receiver nickel complex’s dithiolene ligand was accessible by this approach. Surprisingly, development of [NiFe]-H_2_ase model bimetallic compounds featuring nickel tetrathiolate centers as donors has remained scarce, although the Millar and Maroney groups have prepared stable potential candidates.^45−48^ This lack primarily stems from the inherent challenges that homoleptic thiolate complexes pose, such as their susceptibility to oligomerize or their propensity to uncontrollably bind to multiple metal centers within thermodynamic wells. Our approach involves the utilization of robust NiS_2_ receiver units with (NO)Fe(N_2_S_2_). These units serve as innovative building blocks for engaging metallodithiolates, ultimately furnishing the pivotal bridging dithiolates and thus forming the elusive NiS_4_ unit, in this case NiS_2_S_2_’, where S’ = bridging thiolate sulfur. Aside from the structural significance of such a unit from nickel dithiolenes, it also introduces interesting electrochemical, optical, and magnetic properties.^49−51^

**Figure 4:**
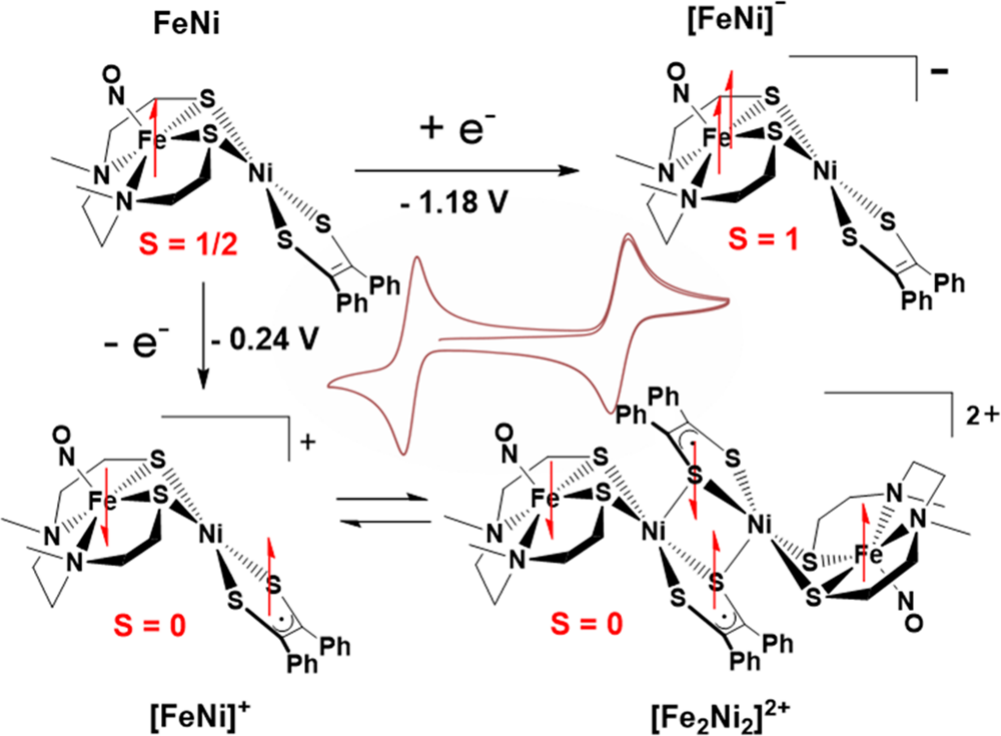
Three redox congeners of the **FeNi** system with radicals denoted as arrows with alignments corresponding to the ground spin states. Inset shows the CV of FeNi displaying two fully reversible redox events. Adapted with permission from ref (2). Copyright 2022 The Author(s). Published by PNAS.

**Figure 5:**
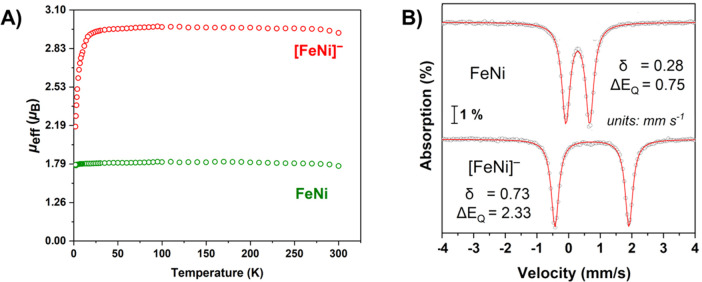
(A) Temperature-dependent magnetic susceptibly data of **FeNi** and [**FeNi**]^−^. (B) Zero-field Mössbauer spectra of **FeNi** and [**FeNi**]^−^ at 5 K. Adapted with permission from ref (2). Copyright 2022 The Author(s). Published by PNAS.

Thus, the sulfur-bridged Fe–Ni bimetallics of this study with NO on iron and dithiolene on nickel were found to produce heterobimetallics with unusual and unexpectedly intricate physical properties.^2^ Good reversibility in two redox events of the as isolated neutral bimetallic complex **FeNi** at −0.24 and −1.18 V led to isolation of oxidized and reduced congeners, respectively; see Figure 4. Characterization by SQUID magnetometry and Mössbauer spectroscopy, Figures 5A and B, informed on the product from reduction of the neutral **FeNi** parent in its anionic form. The spin magnetic moment of 1.74 for the neutral species increased to 2.96 μ_B_ upon reduction and accounted for the *S* = 1/2 to *S* = 1 conversion. The [**FeNi**]^−^ is a rare example of a thermally stable high-spin {Fe(NO)}^8^, found as linear Fe^II^(NO^–^). Mössbauer data are consistent with the redox change at the {Fe(NO)}^7/8^ site with an isomer shift value increase from 0.28 to 0.73 mm s^–1^. A similar difference in isomer shift values was seen in the [Fe*Fe’]^0/–^ compounds.

**Figure 6:**
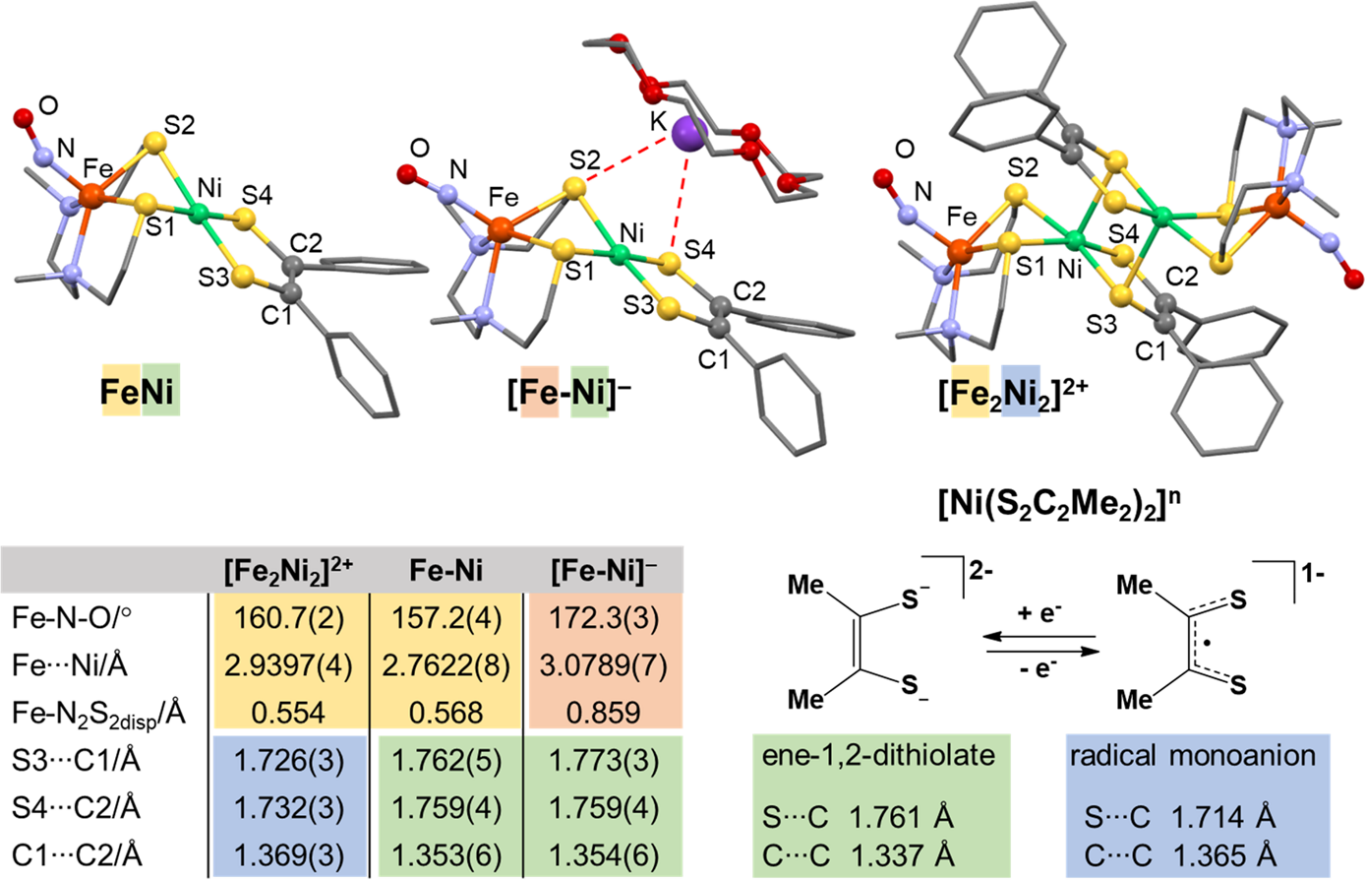
Molecular structures of **FeNi**, [**FeNi**]^−^ and [**Fe**_**2**_**Ni**_**2**_]^2+^, with thermal ellipsoids shown at 50% probability. The hydrogen atoms were omitted for clarity. Structural parameters are tabulated below the structures, and the S–C and C–C distances are compared to the reported [Ni(S_2_C_2_Me_2_)_2_]^*n*^ at two redox levels (*n* = 2– and 1−). Color is used to guide the reader’s view. Adapted with permission from ref (2). Copyright 2022 The Author(s). Published by PNAS.

Oxidation of **FeNi** generated the 2[**FeNi**]^**+**^ ⇌ [**Fe**_**2**_**Ni**_**2**_]^2+^ equilibrium in solution, depicted in Figure 4; crystallization yields only the [**Fe**_**2**_**Ni**_**2**_]^2+^ dimeric dication, isolated as PF_6_^–^ and BArF^–^ salts. Figure 6 displays crucial distinctions in structural characteristics, notably the Fe–N–O angle and the C–C and C–S distances in the dithiolene ligand. In the case of the reduced species [**FeNi**], the most significant changes are observed in the Fe–Ni distance (0.317 Å increase) and hinge angle (18.8° increase). These disparities can be attributed to the Fe displacement from the N_2_S_2_ plane in the anionic form, which was found to be 0.3 Å greater than that found in the cationic and neutral forms. In comparison to the neutral **FeNi** species, the cationic species [**Fe**_**2**_**Ni**_**2**_]^2+^ exhibits a reduction in the S3–C1 and S4–C2 distances within the dithiolene units on Ni, averaging 0.033 Å, while the C1–C2 distance increases by an average of 0.013 Å. Although these changes are relatively small, they suggest that the oxidized Ni-dithiolene unit assumes the radical monoanion form.^52^ Conversely, in the reduced species, where the additional electron is located on the Fe(NO) moiety (*vide infra*), negligible changes are observed in the Ni-dithiolene unit. The increased negative character in the {Fe(NO)}^8^ unit is consequently transmitted to the thiolate sulfurs, resulting in ion pairing between the S4 dithiolene sulfur and the adjacent metallodithiolate S2 sulfur with the K^+^ counterion encased within the 18-c-6 adduct.

**Figure 7:**
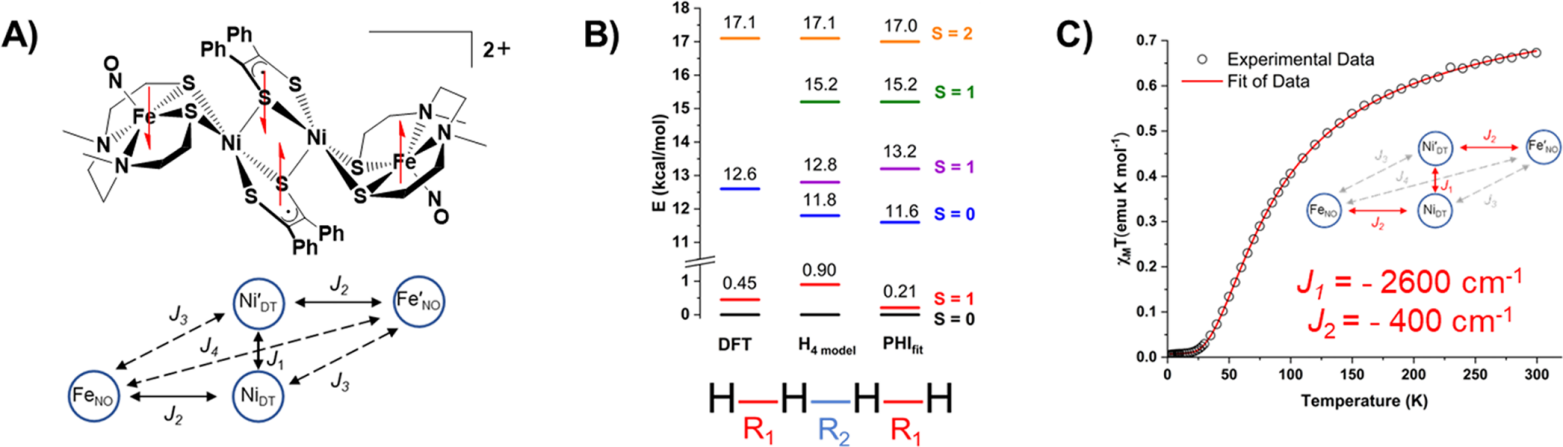
(A) Spin topology of the [**Fe**_**2**_**Ni**_**2**_]^2+^ complex. (B) Energy profiles obtained from DFT, the H_4_ model, and the PHI program. (C) Temperature-dependent magnetic susceptibly data of [**Fe**_**2**_**Ni**_**2**_]^2+^ with fitted parameters obtained from the PHI program. Adapted with permission from ref (2). Copyright 2022 The Author(s). Published by PNAS.

The “monomer” [**FeNi**]^**+**^ appears as a diradical, spin-coupled (and diamagnetic) molecule between {Fe(NO)}^7^ and Ni_DT_^+•^. It could only be studied in solution, while the solid state finds dimerization and coupling of the two Ni_DT_^+•^ via a Ni_2_S_2_ rhomb with anFe–Fe separation of ca. 8 Å. The singlet–triplet gap of monomeric [**FeNi**]^**+**^ was determined using the recent iteration of the variable temperature ^1^H NMR spectroscopy method developed by Brown et al., which was originally described by Cotton and co-workers.^53,54^ Accordingly, we monitored the temperature-dependent behavior of the aromatic phenyl protons on the dithiolene ligand and observed nonlinear changes that were consistent with the population of the excited triplet state. Our data analysis revealed a singlet–triplet gap of approximately 6.9 kcal/mol, corresponding to an exchange coupling constant (*J*) of −1200 cm^–1^ for the antiferromagnetic coupling between the Ni_DT_^+•^ and {Fe(NO)}^7^ radicals. Interestingly, this is ca. half of what is computed for the [Fe_2_(NO)_3_]^+^ cations where direct exchange was observed. As the second radical in the [**FeNi**]^**+**^ is largely localized on the dithiolene ligand, it is reasonable that the coupling is weaker. Magnetic data (SQUID) on the dimer dication, spin topology shown in Figure 7A, found a singlet ground state with a thermally accessible triplet state that is responsible for the magnetism at 300 K (χ_M_*T* = 0.61 emu K mol^–1^). The triplet state was detectable by parallel mode EPR spectroscopy measured in a temperature range of 20–50 K. Hall’s theoretical model built on an H_4_ chain () explains this unexpected ferromagnetic low-energy triplet state arising from antiferromagnetic coupling of a four-radical molecular conglomerate.^2^ The *R*_1_ and *R*_2_ distances were adjusted and benchmarked to match the DFT computed energies (Figure 7B). With both energy profiles the PHI program was used to fit *J*_1_ and *J*_2_, the dominant exchange pathways.^55^ From this model, the radicals involved in the Ni_2_S_2_ rhomb were estimated to be strongly coupled with a *J*_1_ value of −2600 cm^–1^ (Figure 7C). This bridge was thus interpreted to be essentially diamagnetic and facilitated long-range superexchange from the distal irons with a *J* value of −53 cm^–1^.^2^

## 4 Magnetic Coupling between Fe(NO) Spin Probe Ligands through Diamagnetic Ni^II^, Pd^II^ and Pt^II^ Tetrathiolate Bridges (Case C)

**Figure 8:**
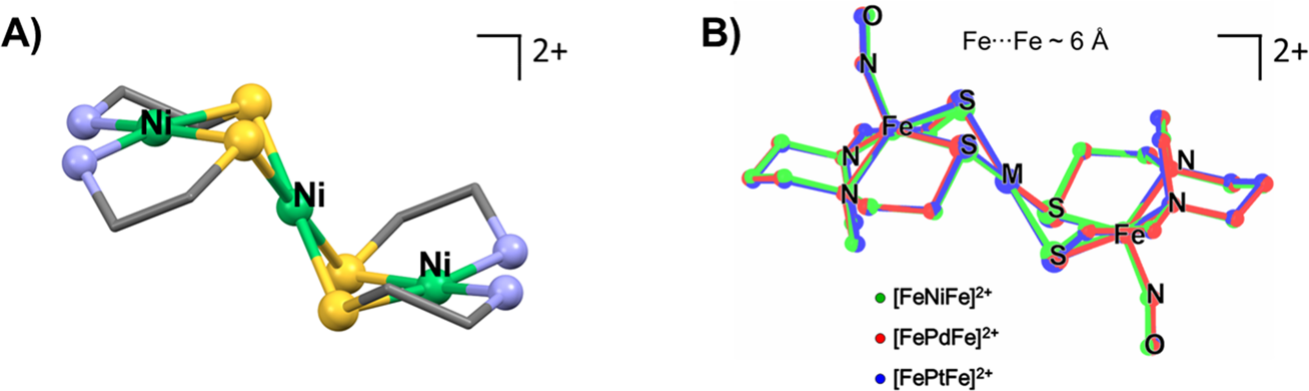
(A) Busch–Jicha complex showcasing the targeted transoid or stair-step arrangement. (B) Structural overlays of the [**FeMFe**]^2+^ complexes from SC XRD of their BF_4_^–^ salts. Adapted with permission from ref (3). Copyright 2023 Royal Society of Chemistry.

In order to benchmark the ability of the Ni_2_S_2_ cluster connector of the (NO)Fe(N_2_S_2_) spin probe to transfer spin coupling information, we explored different bridging units connecting distal Fe(NO) entities. The pioneering examples of metallodithiolates serving as ligands were reported a half century ago, involving the Busch–Jicha complex, formulated as [(Ni(N_2_S_2_)_2_Ni^II^)]^2+^. The structural characterization of this trinickel complex by Dahl and Wei confirmed Busch’s prediction of the chelation of two identical square planar Ni(NH_2_CH_2_CH_2_S)_2_ entities to a third central Ni^2+^ ion (Figure 8A) in a transoid or stair-step arrangement.^56−58^ As this overall topology is similar to or the same as our [**Fe**_**2**_**Ni**_**2**_]^2+^ complex, we anticipated that a useful series could be derived from (NO)Fe(N_2_S_2_) donors and group 10 dications.

**Figure 9:**
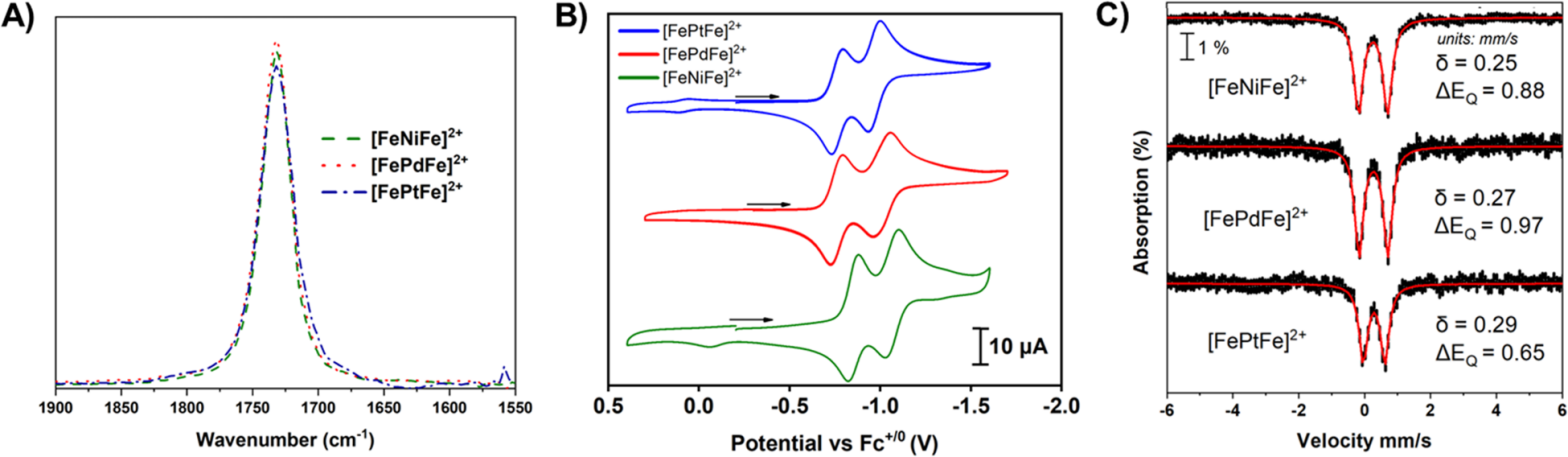
FTIR spectra (A), CVs (B), and Mössbauer spectra (C) of the [**FeMFe**]^2+^ (M = Ni, Pd, Pt) complexes. Adapted with permission from ref (3). Copyright 2023 Royal Society of Chemistry.

Reaction of the nitrosylated-iron metallodithiolate ligand, paramagnetic (NO)Fe(N_2_S_2_), with [M(CH_3_CN)_*n*_][BF_4_]_2_ salts (M = Ni^II^, Pd^II^, and Pt^II^; *n* = 4 or 6) affords diradical trimetallic complexes in the expected stair-step type arrangement ([**FeMFe**]^2+^, M = Ni, Pd, and Pt), with the central group 10 metal held in a MS_4_ square plane, connecting the two spin probe ligands (Figure 8B).^3^ These isostructural compounds have nearly identical ν(NO) stretching values, isomer shifts, and electrochemical properties, as shown in Figure 9.

**Figure 10:**
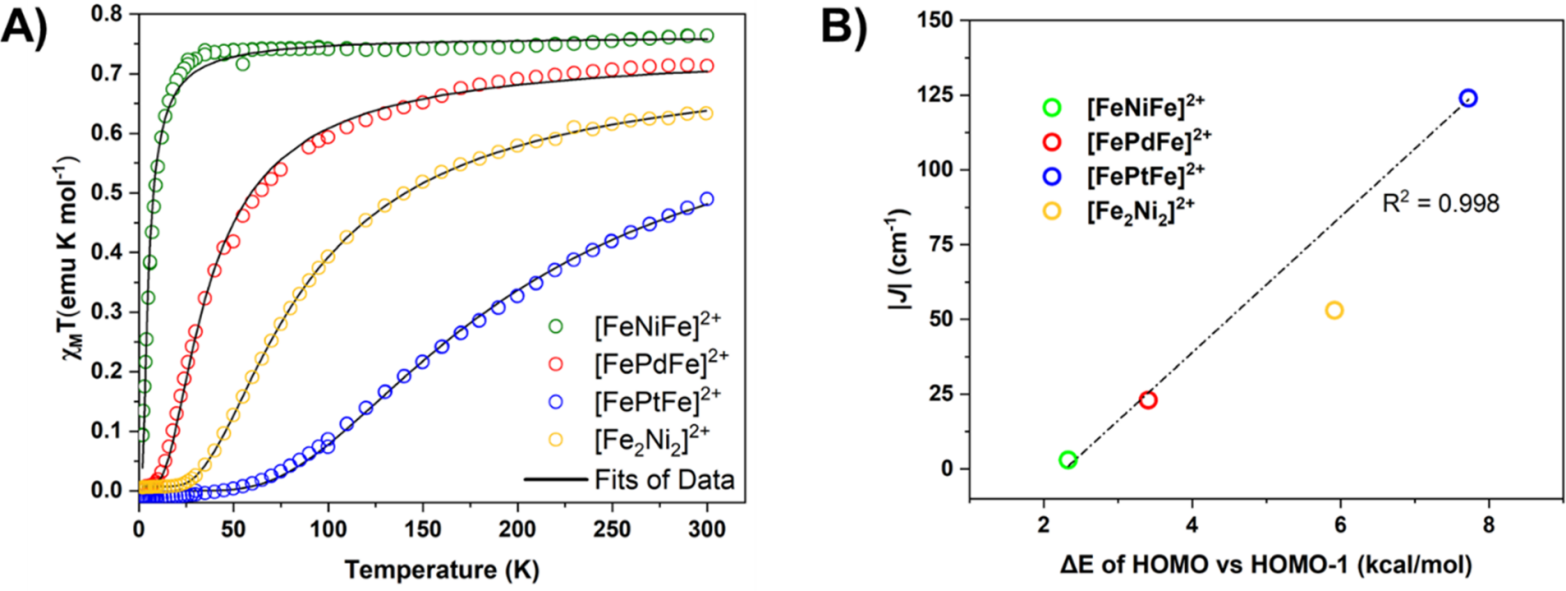
(A) χ_M_*T* vs *T* plots for [**FeNiFe**]^2+^ (green circles), [**FePdFe**]^2+^ (red circles), [**FePtFe**]^2+^ (blue circles), and [**Fe**_**2**_**Ni**_**2**_]^2+^ (yellow circles). Black curves are fits of the experimental data. (B) Linear relationship between experimental *J* values vs the energy gap between the HOMO and HOMO-1 of the triplet states. Both figures adapted with permission from ref (3). Copyright 2023 Royal Society of Chemistry.

Although identical in the physical properties described above, there is a significant difference in their magnetic properties. Despite the intramolecular Fe–Fe distances of ca. 6 Å, variable-temperature magnetic susceptibility measurements (Figure 10A) find antiferromagnetic coupling between {Fe(NO)}^7^ units, with supporting evidence from EPR and DFT studies. The superexchange interaction through the thiolate sulfur and central metal atoms is on the order of Ni^II^ < Pd^II^ ≪ Pt^II^ with exchange coupling constants (*J*) of −3, −23, and −124 cm^–1^, consistent with the increased covalency of the M–S bonds (3*d* < 4*d* ≪ 5*d*). This trend is reproduced by DFT calculations with molecular orbital analysis providing insight into the origin of the enhancement in the exchange interaction. Specifically, the magnitude of the exchange interaction correlates surprisingly well with the energy difference between the HOMO and HOMO-1 orbitals of the triplet states (Figure 10B), which is reflected in the contribution of these orbitals from the central metal. These results demonstrate the ability of sulfur rich metallodithiolate ligands to engender strong magnetic communication by virtue of their enhanced covalency and polarizability.

For comparison, note that the 2-nickel, 2-sulfur cluster bridge in [**Fe**_**2**_**Ni**_**2**_]^2+^ exhibits a 2-fold enhancement in the magnetic coupling between the distal two iron-nitrosyl spin centers compared to that of the palladium bridge; see yellow circles data in Figures 10A and B. We posit that we have exposed a unique example of how Earth-abundant metals, when paired with supporting redox-active centers, can match or even outperform heavier precious metals. In fact, this result underscores the central theme of this Account: sulfur facilitates the close proximity of metals, while π-delocalizing ligands accommodate changes in charge.

## 5 Sulfur Lone Pairs Control Topology (Case D)

**Figure 11:**
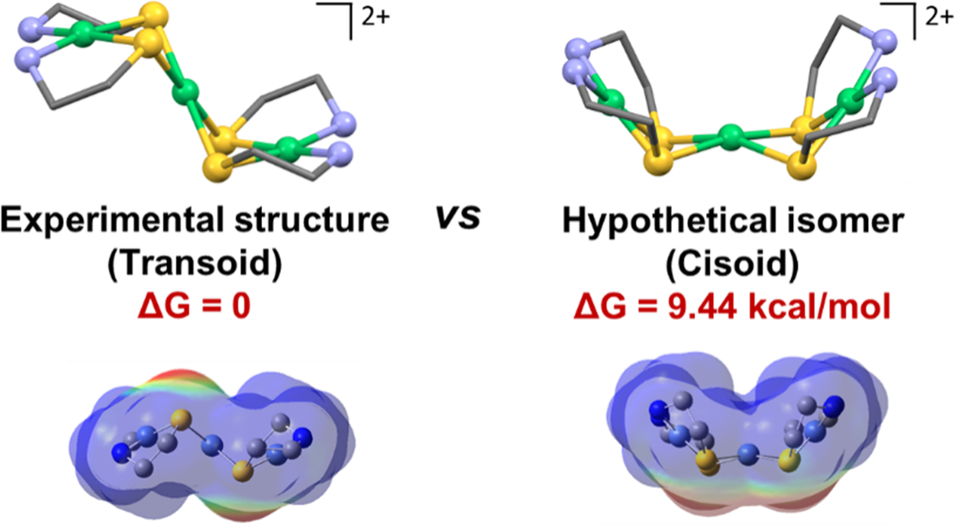
Busch–Jicha experimental structures vs the hypothetical cissoid isomer with the energy difference shown, including ESP maps of each arrangement.

Mentioned above was a tribute to the pioneering work of Daryle Busch concluding that the nucleophilicity of the thiolate sulfurs of NiN_2_S_2_ could template reactions with wide ranging applications or implications, even in bioinorganic chemistry. An interesting question arises regarding the topology of the [Ni_3_]^2+^ complex (Figure 11) and the many others we have added to this class of complexes, including the [Fe(NO)]^2+^ analogues linked by the square-planar-favored group 10 metal cations. As was found for the Busch/Jicha complex by Larry Dahl, experimental structures place the two metallodithiolate ligands in a transoid arrangement, whereas the cissoid arrangement is rarely observed. The DFT-derived free energies indeed find the transoid isomer of the Busch–Jicha trimetallic to be more stable than the hypothetical cissoid by 9.44 kcal/mol. Electrostatic potential maps indicate unfavorable electrostatic repulsion between four thiolate lone pairs for the cissoid arrangement. The “discovery” of the latter isomer to be common in a series of trimetallics designed from the metallodithiolate as ligand approach comprises the fourth member of our case studies.

**Figure 12:**
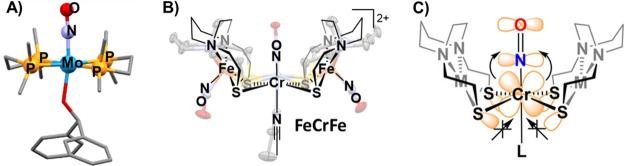
(A) Example of a {Mo(NO)}^6^ diphos complex.^59^ (B) [**FeCrFe**]^2+^ complex overlaid on the corresponding crystal structure. (C) ChemDraw depiction of the residual S-lone pairs and Cr(NO) π-orbitals in the cissoid arrangement. Adapted with permission from ref (4). Copyright 2023 American Chemistry Society.

Established according to the *v*(CO) values of the W(CO)_4_ acceptor probe, the electron-donating properties of the MN_2_S_2_ class of metallo-*cis*-dithiolates (with hydrocarbon N to N and N to S linkers, and featuring metals like Ni^2+^, [Fe(NO)]^2+^, and [Co(NO)]^2+^) are similar to traditional bipyridine or diphosphine ligands. In search of different arrangement centers, we noted that synthetic efforts from the Richards and Louttit groups had produced a series of diphosphine ligands bound to {Mo(NO)}^6^ and {Cr(NO)}^5^ in a trans-configuration (Figure 12A).^59,60^ Similar approaches with the Ni(N_2_S_2_) and (NO)Fe(N_2_S_2_) complexes as ligands found that the thermodynamically stable trimetallic products linked with {Cr(NO)}^5^ were in the cissoid arrangement; the[(NO)Fe(N_2_S_2_)]_2_Cr(NO)(CH_3_CN)]^2+^ or [**FeCrFe**]^2+^ product is shown in Figure 12B. The unique stereochemical behavior found in the “uncommon” isomer emerges from the additional lone pair(s) interactions on the sulfur donor atoms.^4^

The difference when the central nickel atom in the Busch–Jicha complex structure is replaced by {Cr(NO)}^5^, a paramagnetic (*S* = 1/2) dication bridge, is not only in the cissoid arrangement but also in a seemingly minor but reproducible metric parameter from XRD data. Within this strong S_4_ π-bonding framework, chromium rises out of the S_4_ plane. This distortion results from orientation of the four residual S-lone pairs toward the same *d* orbitals that participate in π-bonding with NO. Consequently, the antibonding interaction shown in Figure 12C finds stabilization of the system through engaging the delocalization effects of the NO. Thus, in comparison to the four phosphorus donors, the extra lone pairs of the four sulfur donors favor the distinctive cissoid structure through synergic interactions with the nitrosyl ligand.

Interestingly, the ν(NO) value of 1734 cm^–1^ for the Fe(NO) of the cissoid [**FeCrFe**]^2+^ complex is identical to other [**FeMFe**]^2+^ complexes (in transoid topology). The Mössbauer spectrum also has a similar δ value of 0.27 mm/s, however, with a greater Δ*E*_Q_ of 1.00 mm/s. The significant difference in Δ*E*_Q_ suggests changes in the ligand environment at the Fe center as the bidentate S-donors are modulated by the receiver metal ion, resulting in a slight difference in the ligand field that can be attributed to the difference in sulfur interactions in the cissoid arrangement.

**Figure 13:**
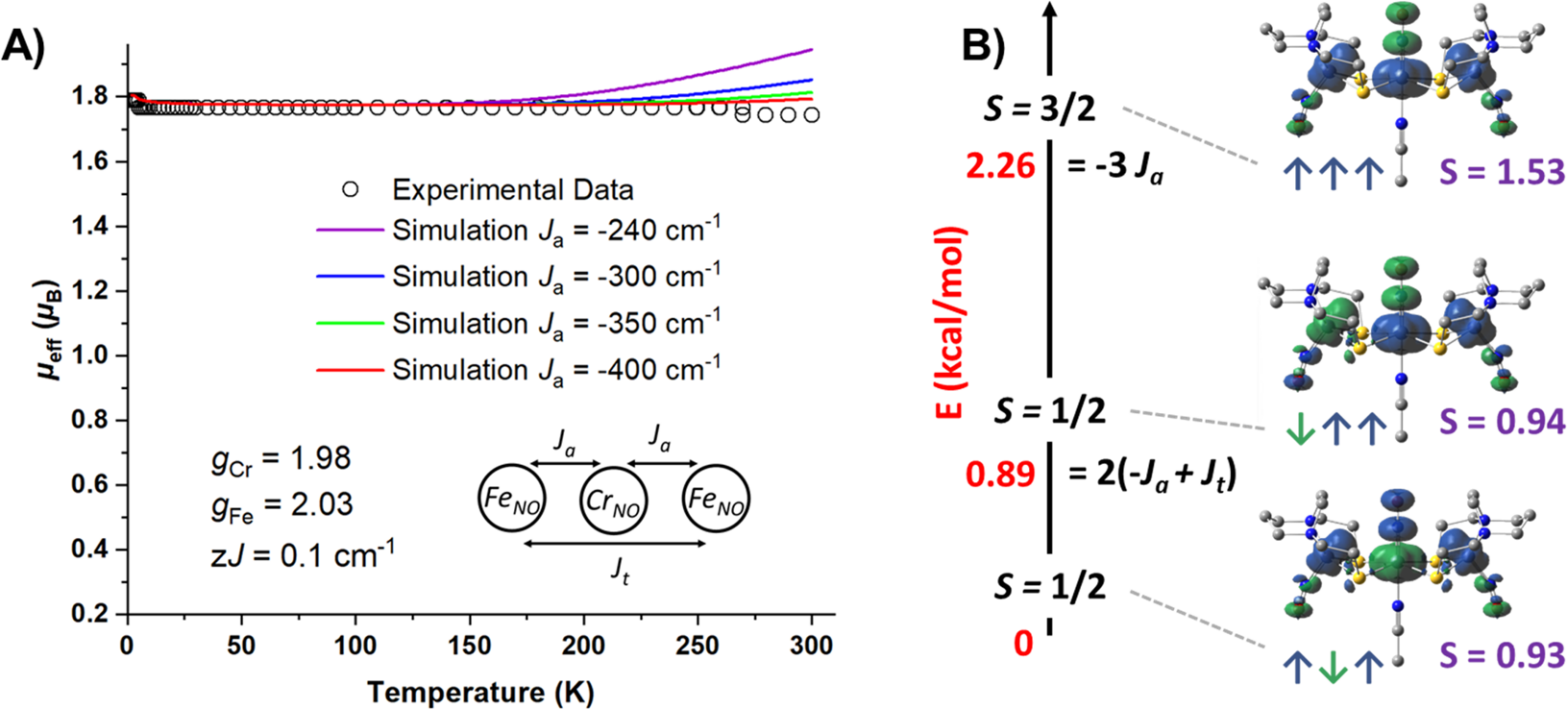
(A) Simulations of the μ_eff_ vs *T* data of [**FeCrFe**]^2+^ with *g* values of Cr and Fe fixed while varying *J*_a_. (B) DFT calculated spin ladder of [**FeCrFe**]^2+^ with the corresponding energies, equations, spin density plots with spin alignments, and computed *S* values shown for each spin state.

The spin topology for a trinuclear system whose spin centers are in a linear arrangement is shown in the inset of Figure 13A. This model includes coupling between adjacent metal centers, *J*_a_, and coupling with terminal metal centers, *J*_t_. This exchange coupling leads to three spin states composed of one *S* = 3/2 and two *S* = 1/2 states that can be expressed in symbolic fashion as (↑↑↑) for the former states and (↑↑↓) or (↑↓↑) for the latter states. In our case, the energies are given by E(↑↓↑) = 0 for the ground state, E(↑↑↓) = 2(−*J*_a_ + *J*_t_) for excited doublet state, and E(↑↑↑) = −3*J*_a_ for the excited state quartet (Figure 13B). Note that the quartet state only depends on *J*_a_ and only lowering of the energy of this state leads to an increased μ_eff_ at higher temperatures. The [**FeCrFe**]^2+^ complex exhibits a temperature-independent effective moment of 1.76 μ_B_ corresponding to a *S*_t_ = 1/2 ground state with no thermal accessibility to the quartet state. Since the *g* values of {Fe(NO)}^7^ and {Cr(NO)}^5^ are well-known to be 2.03 and 1.98, respectively, they can be fixed with the addition of a *zJ*’ value of 0.1 cm^–1^ to account for very weak intermolecular interactions at ∼4 K. Simulations of the [**FeCrFe**]^2+^ data were carried out with these fixed parameters as shown in Figure 13A, indicating that *J*_a_ is at least −400 cm^–1^. It is documented that meaningful values of *J*_t_ are essentially unattainable when |*J*_a_| ≫ |*J*_t_|; however, through DFT computations an estimate of *J*_t_ (Fe–Fe coupling) can be obtained.^61,62^ The full spin ladder of [**FeCrFe**]^2+^ was calculated revealing that the excited state doublet (↑↑↓) and quartet states (↑↑↑) are 0.89 and 2.26 kcal/mol, respectively, higher than the ground state doublet (↑↓↑); see spin density plots in Figure 13B. Solving for *J*_t_ and *J*_a_ with the equations discussed above gives values of −263.3 and 24 cm^–1^, respectively. The adjacent coupling is weaker than suggested from simulations of the experimental data; however, the calculated *S* values are highly spin contaminated for both doublets (*S* = 0.9 for both). That is, if the spin is pure (*S* = 0.5), the energy gap between the quartet and doublets would be larger and would give a stronger spin projected coupling constant. Nevertheless, the sign of the spin unprojected *J*_t_ value will remain positive, indicating that the Fe–Fe coupling is ferromagnetic opposite to what was seen for the transoid trimetallic complexes in the previous sections. The magnetic results of [**FeCrFe**]^2+^ showcase that having a paramagnetic bridge gives a well isolated nonzero ground state with ferromagnetic coupling of the distal irons. The former implication is of great interest since well isolated ground states due to strong superexchange are a desired feature in the design of small molecular magnets.

## 6 Postscript: Both Donors and Receivers Are Important

**Figure 14:**
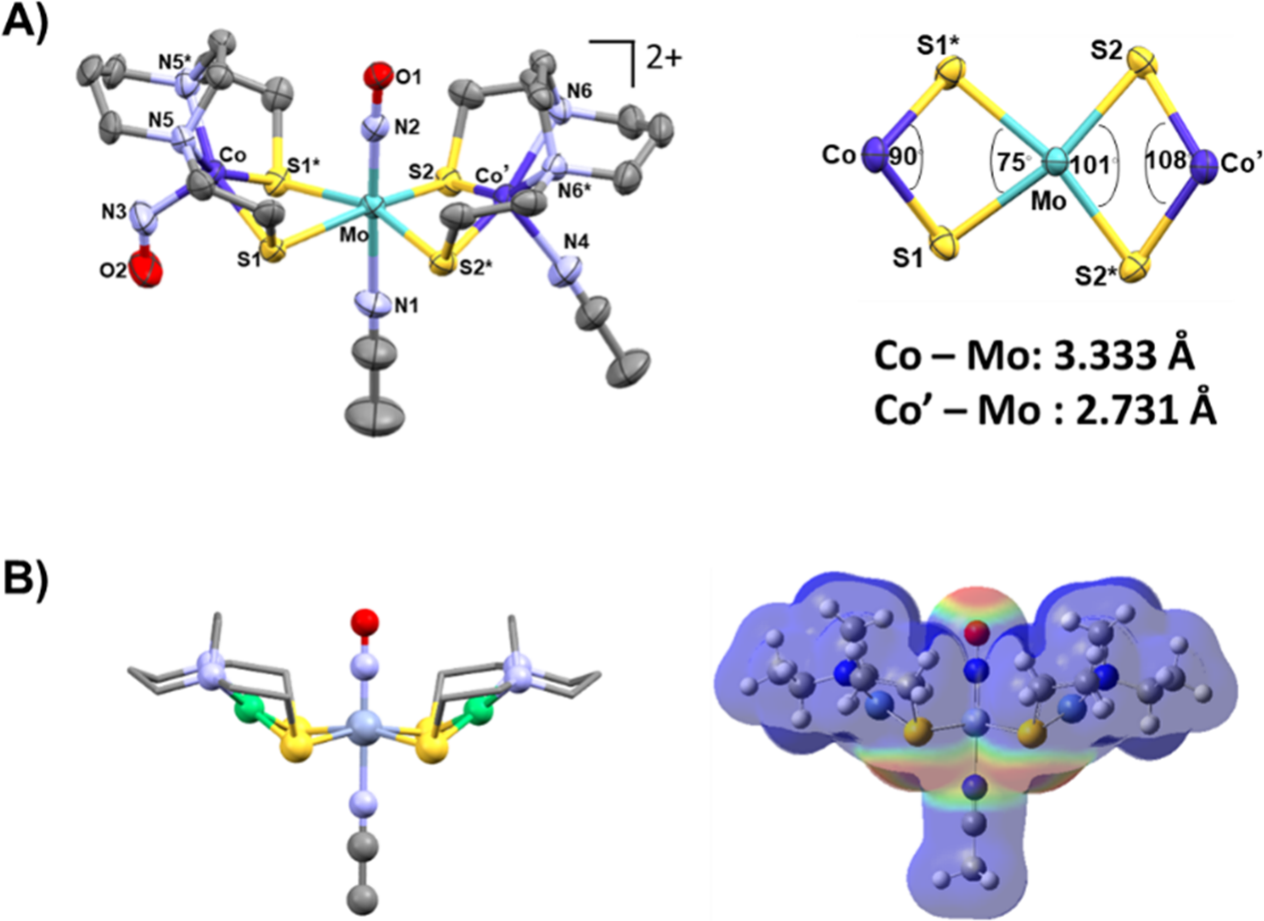
(A) Asymmetric [**CoMoCo’**]^2+^ trimetallic complex showing the differences in metric parameters for the Co and Co’ fragments. (B) Optimized structure of [**NiCrNi**]^2+^ with its ESP map shown in a similar topology. Adapted with permission from ref (4). Copyright 2023 American Chemistry Society.

A postscript to this section is in regard to the molybdenum analogue of the Cr(NO) bridge and the possibility that the metallodithiolate ligands are not always chemically innocent. Attempts to prepare the FeMoFe derivative analogous to the Cr trimetallic led to oxidation of the system, which settled into the Fe_2_(NO)_3_ cation thermodynamic valley that was described in the first section of this Account. Hence, we turned to the (NO)Co(N_2_S_2_) as a metallodithiolate ligand with the result of a [**CoMoCo’**]^2+^ complex as indicated in Figure 14A.^63^ By two synthetic routes, the cobalt nitrosyl finds thermodynamic stability in which one metallodithiolate ligand is largely as it is in the free ligand, whereas the second has transferred its NO ligand to the molybdenum synthon, and an asymmetric butterfly topology results. The second cobalt dithiolate opens the S–Co–S angle to ca. 108° compared to the 75° of the unchanged ligand. Molecular orbital computations (NBO analysis) find the differences in Co–Mo distances of 3.333 Å vs 2.731 Å to reflect a one-electron bond in the complex with shorter metal–metal distance. Notably in both the Cr and Mo versions, with Ni-, Co- or Fe-metallodithiolate, the cissoid structures create an electropositive binding pocket for the NO clearly identified in the electrostatic potential plots in Figure 14B.

## 7 Remarks

**Figure 15:**
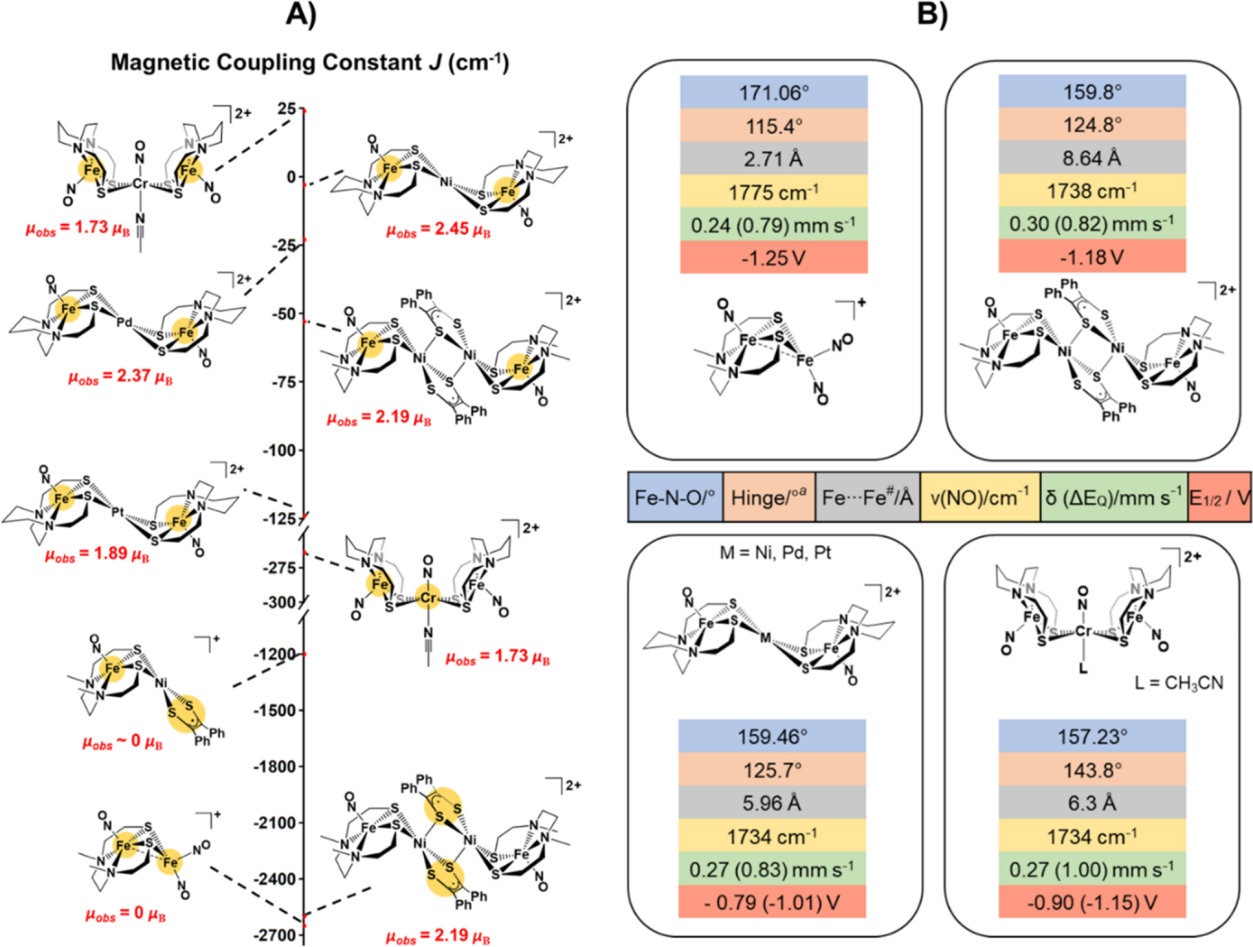
(A) Scale depicting the range, sign, and magnitude of the magnetic coupling constants found for the compounds of this overview. (B) Summary of spectroscopic and structural properties for the compounds of this overview. The hinge angle in this case is the intersection between the two S–M–S planes. FTIR and redox potentials were recorded in CH_3_CN.

The development of specific-purpose ligands has provided numerous significant scientific advances, particularly in the fields of organometallic chemistry related to catalysis (witness the N-heterocyclic carbenes and the Grubbs catalyst), bioinorganic chemistry, and enzyme active sites and the preparation of novel molecules and new inorganic solid-state materials. These ligands are often evaluated based on their impact on the metals they bind to, with a focus on their steric and electronic properties. For instance, as described above, ligands like MN_2_S_2_, which donate four electrons in a bidentate binding mode, share similarities with well-known ligands such as diphos and bipy. By modifications of substituents, the common diphosphines are electronically tunable as sigma donors with variable steric properties as well. The bipyridine class of ligands offers electron delocalizing properties, photo- and redox activity, and the possibility for synthetic designs wherein the binding motif may be incorporated into numerous π-delocalizing organic platforms. The nickel dithiolates inspired by organometallic natural products such as the active sites of hydrogenase and ACS enzymes, and the (NO)Fe(N_2_S_2_) that was the focus of this Account, offer additional unique features, in that they permit one to track changes in electronic structure through distinctive structural features at the iron center, as well as through parameters like ν(NO) values, EPR, the {Fe(NO)}^7/8^ redox couple, ^57^Fe Mössbauer, and magnetic susceptibility. Figure 15 summarizes these physical properties for bi- and polymetallic complexes containing {Fe(NO)}^7^ units of this overview.

Additionally, the structures (topologies) of the heterometallic complexes are influenced by the extra sulfur lone pairs that are not engaged in σ bonding to the attached metal, leading to unique structural/topological arrangements and electronic properties achievable only with extreme synthetic effort with traditional ligands.

Specifically, (NO)Fe(N_2_S_2_) has proven to be a useful tool as a spin probe for studying magnetic coupling interactions, both direct exchange and superexchange. Some key takeaways from our case studies are as follows: (1) The M-(μ-SR)_2_-M structural motif is flexible and able to feature both types of exchange interactions due to the directionality of the sulfur p orbitals. Direct exchange is observed when the M-(μ-SR)_2_-M motif adopts a flat rhomb, as seen in the [**Fe**_**2**_**Ni**_**2**_]^2+^ system, where π spin delocalization within the nickel-dithiolene monoradical aids in the exchange interaction. Similarly, this observation is made when this motif adopts a hinge angle short enough to allow weak metal–metal interactions, as in the [Fe*Fe’]^**+**^ case. (2) In our studies on these types of systems, it appears that as the hinge angle increases, the superexchange interaction decreases in the order of [Fe*Fe’]^**+**^, [**FeNi**]^**+**^, and [**FeCrFe**]^2+^ (adjacent Fe–Cr coupling). (3) Concerning long-range exchange interactions, we found that coupling between Fe(NO) is weakest with Ni^II^ as the central bridge due to poor mixing of orbitals and is improved when using 4*d* or 5*d* group 10 metals. Utilizing a highly spin-delocalized/strongly coupled diradical bridge can greatly enhance the long-range coupling, as seen in [**Fe**_**2**_**Ni**_**2**_]^2+^. (4) The radical bridge Cr(NO) is the only example where ferromagnetic coupling is indicated between the distal Fe(NO). This is due to the spin alignment of the three radicals and corresponding energetics within the spin ladder, which are largely dependent on the adjacent Fe–Cr coupling.

Understanding the intricacies of magnetic spin coupling interactions is essential across a wide range of fields. In the realm of catalysis involving spin-coupled metals, these interactions affect electronic structures and redox properties, likely shaping the reactivity of the system in intricate ways that are difficult to quantify.^64−66^ Introducing spin coupling in lanthanide complexes has proven effective in mitigating the undesirable effects of quantum tunnelling, which can impede the slow magnetic relaxation typically observed in small molecular magnets.^67,68^ These interactions also find practical applications in spintronics and memristor devices that rely on molecular films. In such contexts, spin interactions introduce additional state variables that can respond to external stimuli, such as magnetic fields in spintronics and voltage in memristors.^69,70^ As a result, spin coupling has become an indispensable tool in cutting-edge technologies. We welcome other researchers to explore the full potential of (NO)Fe(N_2_S_2_) and related donors to target the applications mentioned above.

## Supporting Information Available

Comparison of the various (NO)Fe(N_2_S_2_) metallodithiolate ligands with characteristic structural parameters and ν(NO) values (PDF)
