# Supplementary material for: Development of (NO)Fe(N2S2) as a Metallodithiolate Spin Probe Ligand: A Case Study Approach
Source: Acc Chem Res. 2024 Feb 28;57(6):831–44. doi: 10.1021/acs.accounts.3c00667 (PMC10979402; doi:10.1021/acs.accounts.3c00667)
Supplement: Supplementary file 1 — ar3c00667_si_001.pdf [file ar3c00667_si_001.pdf]

Supporting Information for

Development of (NO)Fe(N_2S_2) as a Metallodithiolate Spin Probe Ligand: A Case Study Approach

Manuel Quiroz and Marcetta Y. Darensbourg*

Department of Chemistry, Texas A & M University, College Station, Texas 77843, United States

Email: marcetta@chem.tamu.edu*

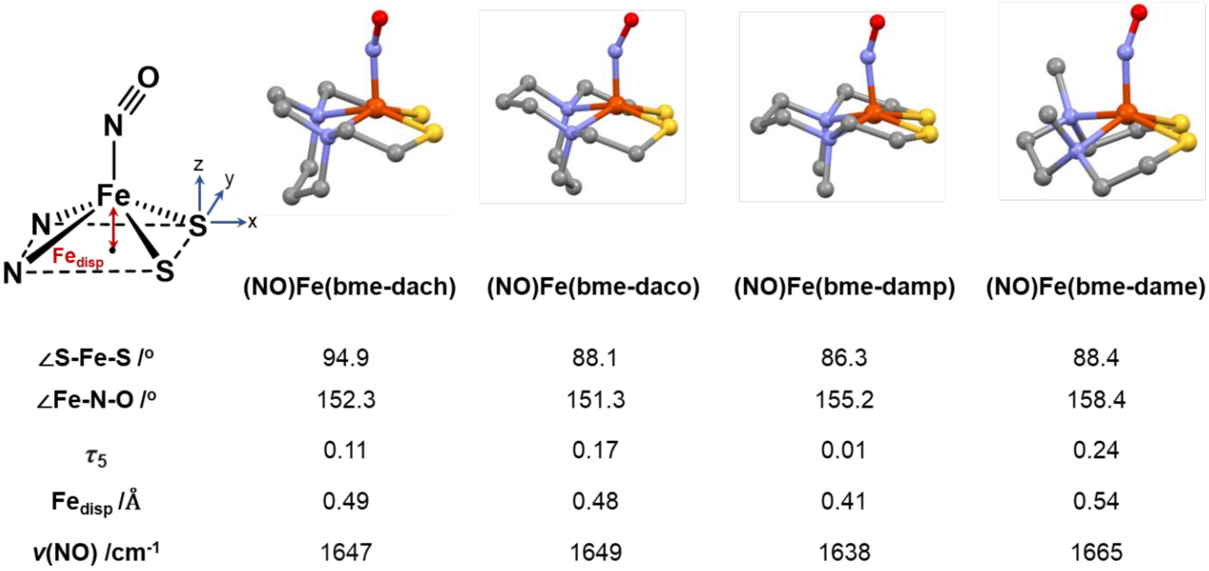

	(NO)Fe(bme-dach)	(NO)Fe(bme-daco)	(NO)Fe(bme-damp)	(NO)Fe(bme-dame)
∠S-Fe-S /°	94.9	88.1	86.3	88.4
∠Fe-N-O /°	152.3	151.3	155.2	158.4
τ_5	0.11	0.17	0.01	0.24
Fe_{disp} /Å	0.49	0.48	0.41	0.54
ν(NO) /cm^{-1}	1647	1649	1638	1665

Figure S1. Various (NO)Fe(N_2S_2) metallodithiolate ligands with characteristic structural parameters and ν(NO) values in CH_2Cl_2.
